# Prioritizing SARS-CoV-2 testing in a highly immunosuppressed patient population

**DOI:** 10.1017/ash.2022.74

**Published:** 2022-05-16

**Authors:** Jenna Shackelford, Michele Woolbert, Ninet Sinaii, Brooke Decker, Tara Palmore, Robin T Odom

## Abstract

**Background:** The NIH Clinical Center implemented multiple testing protocols to facilitate early detection and isolation of SARS-CoV-2 infected patients and rooming-in family members (RIFMs). Beginning in February 2020, all symptomatic patients were tested; in March 2020, all patients were tested prior to aerosol-generating procedures (AGPs); and in May 2020, all patients and RIFMs were tested on admission. We sought to determine the value of SARS-CoV-2 testing practices in our hospital. **Methods:** Respiratory specimens collected March 2020 through June 2021 tested for SARS-CoV-2 by RT-PCR were reviewed, and corresponding patient clinical and demographic variables were collected. Repeated tests from SARS-CoV-2–positive persons were excluded from the data. Results associated with multiple testing indications were assigned the highest priority reason based on a predetermined hierarchy. Data were analyzed using the χ^2^ test and logistic regression. **Results:** Of 12,706 results from 5,704 patients, primary testing reasons were pre-AGP (n = 5,387, 43.0%), admission (n = 2,733; 21.8%), and symptomatic testing (n = 2,701; 21.6%). Overall, 159 tests (1.25%) were positive for SARS-CoV-2. Asymptomatic patients tested on admission were 1.8 times more likely to be positive than outpatients tested for any reason (*P* = .003) and 4.2 times more likely than asymptomatic inpatients tested prior to AGP (*P* = .003). Within asymptomatic pre-AGP testing, there was no difference between inpatients (0.46%) and outpatients (0.65%). Hispanic patients were 1.9 times more likely to be positive. (p **Conclusions:** At a hospital with a geographically broad referral base, admissions COVID-19 testing was far more fruitful than pre-AGP testing of inpatients. Pre-AGP used the most testing resources yet had the lowest yield. Admissions testing remains beneficial regardless of community transmission rates, while testing prior to AGP could be pared back when community rates of COVID-19 are low and redeployed when community rates rise. **Conclusions:** Our findings that Hispanic persons had higher risk and that transplant patients had lower risk of testing positive suggests differences in the extent to which each subgroup may have been able to shelter from COVID-19 in the community during this earlier phase of the pandemic. Keeping immunocompromised patients safe from COVID-19 while they undergo longitudinal care involves layered precautions in the hospital and in the community that must evolve in response to evidence and epidemiological trends.

**Funding:** None

**Disclosures:** None

